# Understanding molecular mechanisms of vertebral number of variations on Mongolian sheep using candidate genes analysis

**DOI:** 10.5713/ab.24.0212

**Published:** 2024-08-26

**Authors:** Chimgee Purev, Huiguang Wu, Khosbayar Lkhagva, Odbayar Tumendemberel

**Affiliations:** 1 Technology Incubator, Mongolian Academy of Sciences, Ulaanbaatar 13330, Mongolia; 2 College of Veterinary Medicine, Yangzhou University, Yangzhou, Jiangsu 225100, China; 3 Joint International Research Laboratory of Agriculture and Agri-Product Safety, the Ministry of Education of China, Yangzhou University, Yangzhou, Jiangsu 225100, China; 4 Jiangsu Co-Innovation Center for Prevention and Control of Important Animal Infectious Diseases and Zoonoses, Yangzhou University, Yangzhou, Jiangsu 225100, China; 5 Laboratory of Molecular Biology, Mongolian National Center of Livestock Genebank, Khongor soum, Darkhan-Uul 45000, Mongolia; 6 Fish Genetics Laboratory, Pacific States of Marine Fisheries Commission and Idaho Department of Fish and Game, Eagle, ID 83616, USA; 7 Conservation Genetics Laboratory, Boise State University, Boise, ID 83725, USA

**Keywords:** Extra Vertebrae, Genetic Association, Mongolian Sheep, *VRTN* Gene

## Abstract

**Objective:**

This study aimed to investigate the genetic link between variations in vertebral number and meat production traits, such as body weight and body measurements (body length, body height, heart girth, and shin width) in Mongolian (Bayantsagaan) sheep. Additionally, we examined the association of single-nucleotide polymorphisms (SNPs) in candidate genes, particularly vertnin (*VRTN*), nuclear receptor subfamily 6, group A, member 1 (*NR6A1*), and synapse differentiation-inducing 1-like (*SYNDIG1L*), with vertebral number variations and their potential impact on meat production traits.

**Methods:**

The study involved 220 Bayantsagaan sheep from Bayantsagaan soum, Tov province, Mongolia, including 104 sheep with extra vertebrae group and 116 individuals with typical vertebral number as the control group. Morphological data, including body weight and body measurements, were collected, and genetic samples were obtained. The impact of vertebral number on morphological traits was estimated using a general linear model. The SNPs in the *VRTN*, *NR6A1*, and *SYNDIG1L* genes were sequenced, and their association with vertebral number was analyzed using one-way analysis of variance.

**Results:**

Bayantsagaan sheep with extra vertebrae were, on average, 4.45 kg heavier and exhibited higher variability in body size traits compared to the control group. Four polymorphic sites were identified at the *VRTN* gene, with one polymorphic locus (VRTN1716) showing a significant association with vertebrae number and body size. Sheep with C/C genotype at VRTN1716 locus, had more vertebrae and larger body size compared to other genotypes.

**Conclusion:**

The findings suggest that variations in vertebral number and *VRTN* gene polymorphisms are linked to favorable meat production traits in Bayantsagaan sheep. The identified SNP (VRTN1716) associated with vertebral number and body size offers the potential for marker-assisted selection in breeding programs. These results provide valuable insights into the genetic basis of meat production traits in Bayantsagaan sheep and may contribute to the development of more efficient breeding strategies.

## INTRODUCTION

Understanding the genetic association of extra vertebrae in domestic sheep (*Ovis aries*) is crucial for meat production. The number of vertebrae in livestock is a critical factor in determining their body size and carcass length, which is of utmost importance in the meat production industry [1]. In Mongolia, where the economy heavily relies on livestock herding, the 2023 annual livestock census data reveals a staggering 64.7 million heads of livestock, with sheep accounting for 45.5% or 29.4 million heads [2]. Despite these high numbers, the low profit margin per animal raises concerns both from an economic and environmental perspective. To address this issue, Mongolia has shifted its focus towards effective management practices and genetic improvement strategies [3].

Pioneering research conducted by King et al [4] suggested that extra vertebrae in pigs could substantially increase carcass length by up to 80 mm. Subsequent studies have demonstrated that an extra thoracic or lumbar vertebrae in Mongolian sheep can lead to a notable increase in carcass length of 2.4 cm or 3.5 cm, respectively [5]. These findings emphasize the potential economic benefits of selecting sheep with a greater number of vertebrae in breeding programs.

The vertebrate spine is composed of a series of vertebrae arranged in distinct regions, including cervical, thoracic, lumbar, sacral, and caudal [6]. In sheep, the vertebrae are organized from the neck to the sacral region, with a total of 30 vertebrae (7 cervical vertebrae [C], 13 thoracic vertebrae [T], 6 lumbar vertebrae [L], and 4 sacral vertebrae [S]). Mutations in the thoracolumbar position, such as T14L6 or T13L7, have been observed to be the most common [7]. Variations in the number of thoracic, lumbar, and thoracolumbar vertebrae can be found within certain species, including pigs [8], sheep [6], and humans [9]. For example, modern pig breeds such as Duroc, Landrace, and Large White exhibit an increased number of thoracic and lumbar vertebrae, ranging from 21 to 23 [8]. Similarly, variations have been observed in European sheep breeds, with the Texel breed showing a range of 17 to 21 vertebrae, while Chinese breeds, such as Mongolian sheep have a range of 19 to 21 vertebrae [5,8].

Genetic polymorphisms play a crucial role in understanding animal genotypes and their associations with traits such as productivity, reproduction, and economic factors [10]. Two quantitative trait loci (QTLs) were initially identified on chromosomes 1 and 7 of the *Sus scrofa* species [11]. The vertnin (*VRTN*) gene [12] and the nuclear receptor subfamily 6, group A, member 1 (*NR6A1*) gene have been implicated in the regulation of lumbar vertebrae number [13]. Previous studies suggest that both of the *VRTN* and *NR6A1* genes independently contribute to the number of thoracic and lumbar vertebrae in pigs [14]. Recent research by Zhong et al [15] found a significant association between the polymorphism of the Synapse differentiation-inducing 1-like (*SYNDIG1L*) gene g.82573325C>A and the number of thoracic vertebrae in sheep. Additionally, a single nucleotide polymorphism (SNP) rs414302710: A>C in exon-8 of the *NR6A1* gene has shown potential influence on lumbar vertebral number in sheep [1].

Mongolian sheep are highly prized for their ability to produce high-quality products at a low cost, as well as their remarkable adaptation to harsh natural and climatic conditions. These sheep graze on natural pastures throughout the year, making them a sustainable and resilient choice for livestock herding. Within Mongolian sheep populations, variations in vertebral number are common, and configurations of vertebrae number such as T14L7 (thoracic), T14L6, and T13L7 have been observed in 12.8% to 24.9% of individuals [16]. The Bayantsagaan sheep, the subject of this study, exhibited a high frequency of these variations at 66.6% [17]. The Bayantsagaan sheep stands out among Mongolian indigenous sheep due to its large size and distinct back and tail characteristics. Its economic value primarily comes from meat, fat, and wool.

This study aims to investigate the impact of vertebral number variations on meat production traits, such as body weight length, height, and heart girth in Bayantsagaan sheep. Additionally, we will explore the association between SNP genotyping of candidate genes (*VRTN*, *NR6A1*, and *SYNDIG1L*) and these variations in vertebral number.

## MATERIALS AND METHODS

### Ethical statement

All experimental procedures involving animals were approved by the Animal Ethics Committee of the Mongolian University of Life Sciences, ensuring the welfare and ethical treatment of animals (no. VBR-21-01-17, 27 September 2021).

### Animals and phenotypes

Morphological data and tissue samples (ear punch) were collected from 220 sheep. The sheep were divided into two groups, including 104 individuals with extra vertebrae (T14L7, T14L6, T13L7) as the experimental group and 116 individuals with a typical vertebral number (T13L6) as the control group. The sheep with ages between 0.7 and 5 years were randomly selected and herded under uniform conditions in Bayantsagaan soum, Tuv province in Mongolia between October and November 2021. Phenotypic data were collected, including sex, age, body weight, body length, height at withers, heart girth and shin width.

### DNA extraction and mutation detection

Ear tissue samples were collected from each sheep, preserved in 75% ethanol, and stored at −20°C. Genomic DNA was extracted using the Ezup Column Animal Genomic DNA Extraction Kit (Sangon Bioengineering Co., Ltd, Shanghai, China) according to the manufacturer’s protocol. DNA concentration and integrity were assessed using a NanoDrop 2000 (Thermo Fisher Scientific Inc., Wilmington, DE, USA), and agarose gel electrophoresis. High-quality genomic DNA was defined as having a total volume greater than 50 μL, a concentration higher than 50 ng/μL, and an A260/280 ratio between 1.8 and 2.0.

Candidate genes (*VRTN*, *NR6A1*, and *SYNDIG1L*) hypothesized to be associated with vertebral variation in Bayantsagaan sheep were selected. Reference sequences in GenBank (accession numbers: NC_019464.1, NC_019460.2, and XM_027972017.1) were used for primer design and further analyses. Primer sets were designed using a Geneious Prime (version 2022) [18], or derived from previously published articles (Supplementary Table S1) [19]. Primers were synthesized by Sangon Biotechnology Co., Ltd (China).

Polymerase chain reactions (PCR) were performed using Premix Taq (Ex Taq Version 2.0 plus dye; Takara, Bio Inc., Beijing, China). The specific PCR conditions for each target gene fragments were detailed in Supplementary Table S2. PCR products were checked on 2% agarose gel electrophoresis, and sequenced bidirectionally on the ABI PRISM 3730 sequencer at Sangon Biotech (Shanghai) Co., Ltd (China). Sequence analyses, including base quality checks, alignments, and substitution detections were performed using Geneious Prime (https://geneious.com), DNASTAR (Lasergene version 11.0, https://www.dnastar.com/), and MEGA software (version 11.0) [20,21].

### Prediction of the impact of amino acid substitutions on VRTN protein function

To further elucidate the molecular properties of the *VRTN* gene, we utilized the online tool PROVEAN (Protein Variation Effect Analyzer) [22] to predict the potential impact of amino acid substitutions on the function of the VRTN protein. PROVEAN generates prediction scores based on the alignment of homologous sequences to assess the likelihood of an amino acid substitution affecting protein function. This tool employs a delta alignment score approach, which measures the change in alignment score caused by an amino acid substitution [22]. A default score threshold of −2.5 is used to distinguish between neutral and deleterious variations. Substitutions with a score equal to or below–2.5 are considered deleterious [22].

### Statistical analysis

The relative expression differences of phenotypic traits between the control group with typical vertebrae (T13L6) and the experimental group with extra vertebrae (T14L7, T14L6, T13L7) were analyzed using one-way analysis of variance (ANOVA) with SPSS 19.0 for Windows (SPSS Inc., Chicago, IL, USA) and logistic regression analyses using the binomial generalized linear models (GLM) in R (version 4.3.1) [23]. The results for descriptive statistics in morphological measurements for 220 sheep are analyzed (Supplementary Table S3). We used the full dataset, including these morphological and genetic results in candidate genes for 117 (47 typical and 43 extra vertebrae) of 220 individual sheep, in the further association statistical analyses.

We performed various analyses to assess phenotypic variation, genotype frequencies, polymorphism information content, genetic diversity (*He*), *F_ST_*, and principal coordinate analyses (PCoA) using GenAlEx v6.5 [24], Arlequin [25] and Microsoft Excel 2021. Covariate analysis was performed to identify the independent variables and the variables of interest (e.g., body weights and lengths). We selected the variables with low positive correlations with a threshold (r) of less than 0.50. The variables with low correlated variables, including body weight and body length were chosen for further logistic regression (binomial GLM) analysis.

Two sets of logistic regression models were run to test the morphological significance on extra vertebrae and the statistical significance of each SNP locus, as well as a mixed model using the significant SNP loci and morphological variables to assess their associations with extra vertebrate (Supplementary Table S4–S5). The best model was selected using Akaike’s information criterion [26]. The final logistic regression (GLM models) results, including the confidence intervals, were calculated using the R package “gtsummary” [27].

## RESULTS

### Phenotype association analyses

Our analysis of the phenotypic traits revealed notable differences in means and medians between the 104 sheep with extra vertebrate and 116 typical sheep in the Bayantsagaan sheep population (Supplementary Table S3). The bi-variate correlation coefficients showed that the body length could be a dependent variable, with a low coefficient (r≤0.5) with other variables. The variable body weight was also included in the models due to its importance in meat production (Supplementary Figure S1). In the multifactor analysis of variance, 117 sheep samples were examined after removing the unqualified PCR product sequences, in which 43 individuals had extra vertebrae, while 74 individuals had a typical number of vertebrae. The multifactor analysis on variance revealed that sheep with extra vertebrae were significantly heavier (4.45 kg) and had a longer body (2.8 cm), and heart girth (2.55 cm) compared to sheep with the normal number of vertebrae (Table 1), which is also supported in our logistic regression models with statistically significant differences for body weight and body length in both groups in Bayantsagaan sheep (Table 2).

### Genetic variation and diversity

A 69% success rate was achieved in amplifying and sequencing partial gene sequences of candidate genes: *VRTN* (1,351 bp), *NR6A1* (223 bp), and *SYNDIG1L* (133 bp). No variations were found in *SYNDIG1L*, while 5 SNP loci were identified, including 4 SNP loci in *VRTN* and 1 SNP locus in *NR6A1*. Most loci had low heterozygosity estimates (*He*≤0.02) (Supplementary Table S5), except for VRTN-1716 and VRTN-1917 (*He* = 0.20 to 0.95). The PCoA showed no significant population structure between sheep with extra vertebrae and the control group (Supplementary Figure S2), which might due to the recent human-induced selection for extra vertebrae. The absence of population structure suggests minor genetic differences between the two groups.

### Genotype and phenotype association analyses

Only one locus, VRTN-1716, showed a statistically significant association with extra vertebrae in Bayantsagaan sheep, as evident through multi-way ANOVA (p<0.05) (Table 3) and GLM models (p = 0.029 to 0.005) (Table 4). The C/C genotype, corresponding to a C/T and C/C nucleotide substitution, was more prevalent in the group with thoracolumbar vertebrae compared to the control group with the C/T genotype. Other loci did not demonstrate statistical support for the extra vertebral trait in Bayantsagaan sheep. Mixed logistic regression binomial GLM models combined with statistically significant variables (VRTN-1716 loci, body length, and body weight) showed a significant association with the extra vertebrate trait. In summary, the VRTN-1716 SNP locus is associated with the extra vertebrate, potentially contributing to longer body length (95% confidence interval, 1.01 to 1.12 cm) and larger body (95% confidence interval 1.00 to 1.07 kg) weight in Bayantsagaan sheep.

### Neutral impact of VRTN A571V Substitution

To predict the functional impact of the A571V variant in the VRTN protein, we employed the PROVEAN tool, which utilized 39 homologous sequences of VRTN for analysis. In order to enhance computational efficiency and prediction accuracy, these sequences were clustered into 13 groups by PROVEAN. The A571V variant, which is characterized by the substitution of alanine (A) with valine (V) at position 571 of the VRTN protein, was the focus of our analysis. The PROVEAN results showed a prediction score of 0.438 for the A571V variant. In consideration of the default threshold of −2.5, which differentiates between neutral and deleterious variations [22], our findings indicate that the A571V substitution is likely to have a neutral impact on VRTN protein function.

## DISCUSSION

This study has shed light on the relationship between vertebral number variations and meat production traits in Bayantsagaan sheep. The research revealed that the number of vertebrae plays a significant role in determining body weight, body length, and heart girth. Additionally, the study showed a strong association between SNP genotyping of candidate genes, particularly the vertinin gene (*VRTN*) in the study area.

The superior meat-production performance of sheep with multiple vertebrae compared to normal sheep, exhibiting longer longissimus muscle, larger abdominal cavity volume, higher carcass weight, increased net meat weight, and a higher lean meat percentage [19]. These heritable traits highlight the potential value of understanding the mechanism behind the vertebral number variation for sheep breeding with enhanced meat-production capabilities [5]. By unraveling the molecular intricacies of this trait, we can make informed breeding decisions that will enable the development of sheep breeds with enhanced meat-production capabilities. This research has the potential to greatly benefit the sheep industry and contribute to improve the meat production outcomes.

The observed morphological difference between the experimental and control groups in the Bayantsagaan sheep population support the hypothesis that vertebral number variation impacts overall body conformation. The significant variations in body weight, body length, and heart girth (Supplementary Table S3) align with previous studies conducted on different sheep breeds, emphasizing the influence of genetic factors in morphological traits [28]. In addition, the *SYNDIG1L* gene has been identified as a factor that influence final body weight and backfat thickness in Landrace pigs [29], and plays a pivotal role in shaping the body structure of cattle [30]. These findings highlight the potential significance of the *SYNDIG1L* gene in relation to body conformation across different animal species. By delving deeper into the genetic mechanisms underlying vertebral number variation and its influence on body conformation, we can gain a better understanding of the genetic factors that contribute to these morphological traits. This knowledge can be valuable in breeding programs aimed at enhancing specific traits, such as body weight, body length, and overall body conformation, in livestock species.

Our research found no any variation in the *SYNDIG1L* gene sequencing in Bayantsagaan sheep. In contrast, with the previous studies by Zhong et al [15] found that the polymorphisms of *SYNDIG1L* gene at the position g.82573325C>A were associated with multiple thoracic vertebrae traits in both Small-tailed Han sheep and Sunite sheep. Nevertheless, our multifactor analysis of variance supported the presence of phenotypic differences between the groups, indicating that variation in vertebral number significantly influences traits such as body size, aligning with the research conducted by Zhang et al [19]. The contrasting results regarding the *SYNDIG1L* gene highlight the complexity of genetic mechanisms underlying vertebral number variation and its impact on phenotypic traits. Further investigation and replication of studies are necessary to gain a more comprehensive understanding of these relationships and their implications for breeding programs and livestock management.

Using logistic regression reinforces the association between vertebral number variation and body conformation traits. Our mixed model analysis showed that body length exhibited strong statistical support for its association with the extra vertebral phenotype (Supplementary Table S3). The association of body weight and vertebral number is less conclusive, although ANOVA suggests that weight differences have biological relevance. These results are consistent with the selective breeding for meat production traits in Bayantsagaan sheep and similar findings in other sheep populations [19].

The association analysis revealed a statistically significant relationship between the VRTN-1716 SNP locus and the presence of extra vertebrae (Supplementary Table S6–S7), aligning with previous research that highlighted the role of the *VRTN* gene in vertebral development [31]. It further emphasizes the role of the *VRTN* gene in determining vertebral number in livestock.

Our findings also demonstrated the importance of *VRTN* gene polymorphism, particularly SNP 1716, in relation to thoracic vertebral number (TVN), suggesting that this locus could serve as a potential marker for assessing its impact on thoracolumbar vertebral number [19], which is consistent with the previous studies [31,32].

PROVEAN is a well-established computational tool for predicting the functional impact of amino acid substitutions. In the case of the A571V variant in the VRTN protein, PROVEAN predicted a neutral impact on protein function. However, this tool has certain limitations, particularly in identifying beneficial or gain-of-function mutations [22]. Therefore, this prediction should be interpreted with caution, as the tool may not fully account for the intricate interactions and structural alterations that could influence protein function.

To determine the functional consequences of the A571V variant on VRTN protein activity and its potential physiological implications, experimental validation is indispensable. *In vitro* functional assays, such as enzymatic activity measurements or protein-protein interaction studies, could offer valuable insights into the biochemical properties of the A571V variant. Furthermore, in vivo studies using animal models or cell-based systems could shed light on the biological significance of this substitution in the context of the organism’s physiology and development. Future research efforts should aim to integrate computational predictions with experimental data to obtain a more comprehensive understanding of the role of the A571V variant in VRTN protein function. This integrative approach can provide a more robust assessment of the functional impact of the variant and its potential involvement in related physiological processes.

## CONCLUSION

In summary, our study provides new insights by identifying the association between the VRTN-1617 SNP locus and the extra vertebral trait in Bayantsagaan sheep, which is related to greater body length, body weight, and heart girth. Future research, such as genome-wide association studies, may reveal additional potential loci that influence the unique trait of having multiple vertebrae in Bayantsagaan sheep. It would be beneficial to expand the analysis beyond the Bayantsagaan soum in central Mongolia, where herders selectively breed many sheep with extra vertebrae, to include other sheep populations and breeds. This broader approach can provide further insights into the genotype-phenotype mechanism underlying this trait.

## Figures and Tables

**Table 1:** Morphological significant variables based on multi-factor analysis of variance using one-way analysis of variance (MFAV) on 117 sheep sequences

Phenotype	N	Body length (cm)	Body weight (kg)	Height at withers (cm)
Control group	74	74.513±1.415^*^	63.171±1.468^**^	93.075±1.122^*^
Experimental group	43	77.313±1.661^*^	67.626±1.722^**^	95.623±1.316^*^

Phenotypic values were shown in estimated marginal means±standard error.

* Indicates significant difference between groups (p≤0.05).

** Indicates highly significant difference between groups (p≤0.01).

**Table 2:** Summary regression results from the top model (extra vertebrate ~ body length (BL) + weight (BW) + height (BH)) on the phenotypic variables associated with the extra vertebrate characteristics from Bayantsagaan sheep in Mongolia

Characteristic	Odds ratio	95% Confidence interval	p-value
Body length (cm)	1.07	1.03 to 1.12	0.001
Body weight (kg)	1.07	1.03 to 1.12	0.001
Height at withers (cm)	0.94	0.89 to 1.00	0.053

**Table 3:** Association analysis between VRTN gene candidate SNPs and the number of thoracolumbar vertebrae

Gene	Candidate SNPs loci	Genotype	N	TLVN±SE	p-value
*VRTN*	SNP1712	CC	113	19.372±0.046	>0.05
		CT	4	19.250±0.250	
	SNP1716 ^**^	CC	90	19.433±0.053	<0.05
		CT	27	19.148±0.070	
	SNP1917	GG	111	19.370±0.046	>0.05
		GT	2	19.000±0.000	
		AG	4	19.500±0.289	

SNPs, single-nucleotide polymorphisms; TLVN, thoracolumbar vertebral number; SE, standard error.

** Indicates SNPs that significantly affect the number of thoracic and lumbar vertebrae (p<0.01).

**Table 4:** Summary regression results from the top model (Extra vertebrate ~ VRTN 1716 + body length + body weight) on the VRTN loci and phenotypic variables as additive variables for the association with the extra vertebrate characteristics from Bayantsagaan sheep in Mongolia

Characteristic	Odds ratio	95% Confidence interval	p-value
VRTN1716	-	-	-
CC	-	-	-
CT	0.24	0.08 to 0.62	0.005
Body length (cm)	1.06	1.01 to 1.12	0.029
Body weight (kg)	1.03	1.00 to 1.07	0.075

VRTN, vertnin.

The results are calculated using the R package, “gtsummary” (Sjoberg et al [27]).

## SUPPLEMENTARY MATERIAL

**Supplementary Figure S1.** Bi-Variate Correlation Coefficients between the phenotypic variables in Bayantsagaan Sheep in Mongolia.

**Supplementary Figure S2.** Principal Coordinates Analyses of Bayantsagaan sheep (n = 152) in Mongolia based on the 3 candidate genes’ SNP loci.

**Supplementary Table S1.** The primer information for polymerase chain reactions

**Supplementary Table S2.** Polymerase chain reactions’conditions for amplification of different gene

**Supplementary Table S3.** Descriptive statistics analyses on the body size/body weight in sheep with multiple vertebrae and sheep with normal vertebrae

**Supplementary Table S4.** The list of logistic regression models using binomial generalized linear models ran on phenotypic data to check the association associated with the extra vertebrate characteristics from Bayantsagaan sheep in Mongolia

**Supplementary Table S5**. Genetic parameters of candidate loci in Bayantsagaan Mongolian sheep population

**Supplementary Table S6.** The list of logistic regression models run on VRTN loci and the phenotypic data to check the association associated with the extra vertebrate characteristics from Bayantsagaan sheep in Mongolia

**Supplementary Table S7.** Summary logistic regression results from the top model (Extra Vertebrae ~ VR TN1716 + Body Length + Body Weight) on the VRTN loci and phenotypic variables as additive variables for the association with the extra vertebrate characteristics from Bayantsagaan sheep in Mongolia
