# Supplementary material for: Understanding molecular mechanisms of vertebral number of variations on Mongolian sheep using candidate genes analysis
Source: Anim Biosci. 2024 Aug 26;38(2):247–54. doi: 10.5713/ab.24.0212 (PMC11725747; doi:10.5713/ab.24.0212)

Supplementary materials for the manuscript entitled:

“Understanding Molecular Mechanisms of Vertebral Number of Variations on Mongolian Sheep Using Candidate Genes Analyses”

Authors: Chimgee Purev, Huiguang Wu, Khosbayar Lhagvaa, Odbayar Tumendemberel

Supplementary Figure 1. Bi-Variate Correlation Coefficients between the phenotypic variables in Bayantsagaan Sheep in Mongolia. The abbreviations represent the following body measurements: body weight (BW), body length (BL), height at withers (BH), chest circumference/heart girth (CC), and shin width (SW)

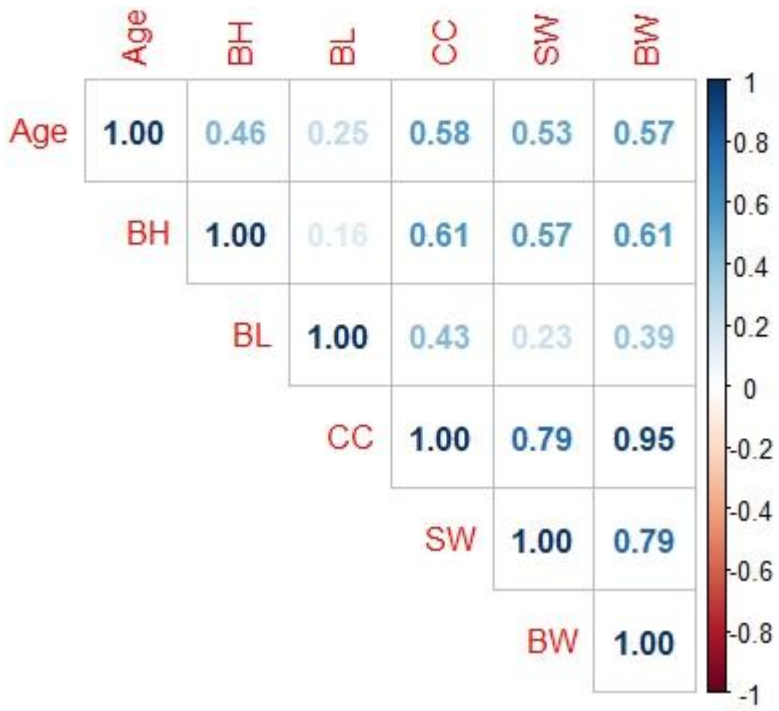

Supplement: Supplementary file 1 [file ab-24-0212-Supplementary-Fig-1.pdf]
