# Supplementary material for: Understanding molecular mechanisms of vertebral number of variations on Mongolian sheep using candidate genes analysis
Source: Anim Biosci. 2024 Aug 26;38(2):247–54. doi: 10.5713/ab.24.0212 (PMC11725747; doi:10.5713/ab.24.0212)

Supplementary Figure 2. Principal Coordinates Analyses of Bayantsagaan sheep (n=152) in Mongolia based on the 3 candidate genes' SNP loci. The control group (1) is shown in orange, and the experimental extra vertebrate group (2) is shown in blue. Each dot represents several samples that have similar genetic variations. There is no indication of population structure based on the 3 candidate genes data.

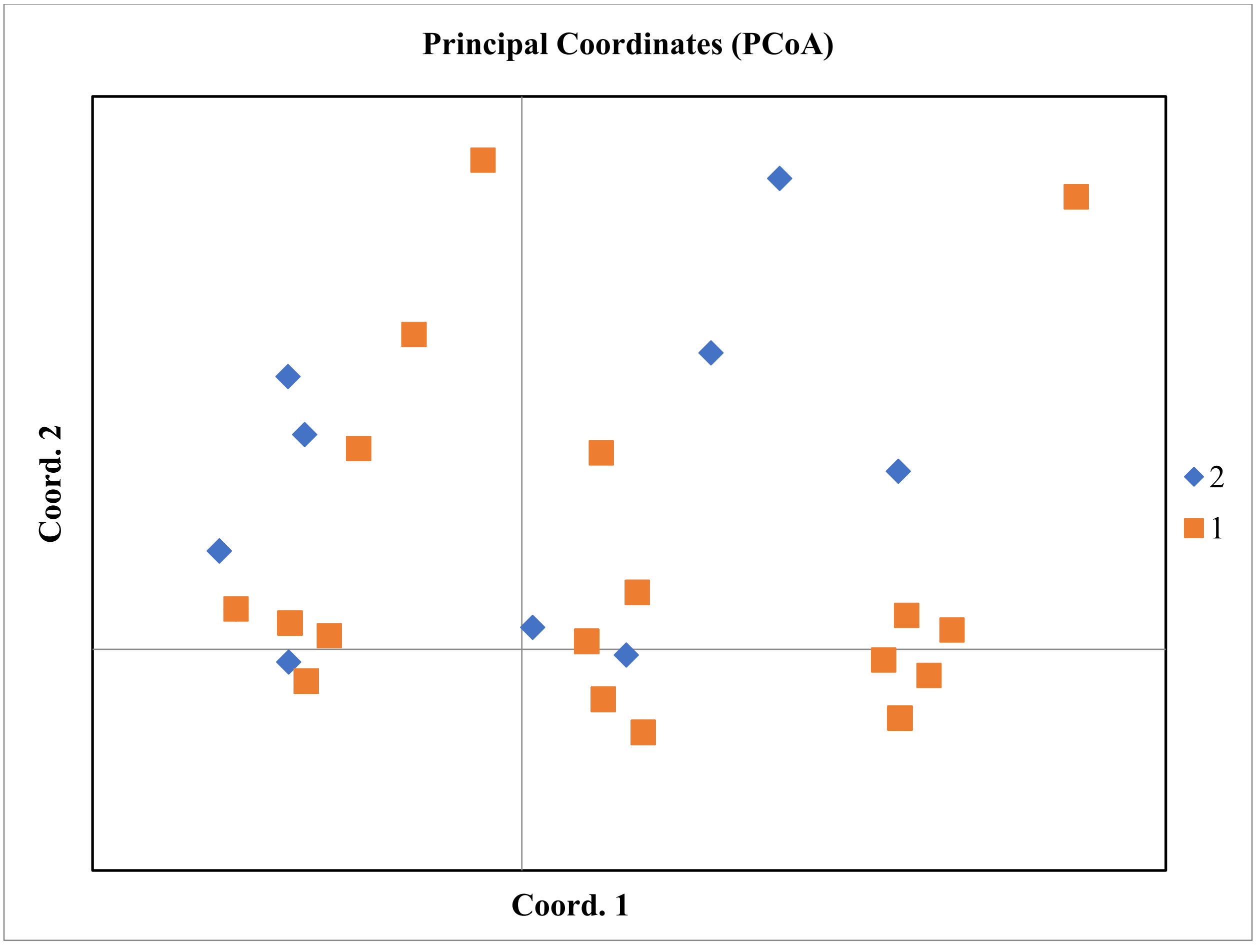

Supplement: Supplementary file 2 [file ab-24-0212-Supplementary-Fig-2.pdf]
