# Supplementary material for: Understanding molecular mechanisms of vertebral number of variations on Mongolian sheep using candidate genes analysis
Source: Anim Biosci. 2024 Aug 26;38(2):247–54. doi: 10.5713/ab.24.0212 (PMC11725747; doi:10.5713/ab.24.0212)
Supplement: Supplementary file 3 [file ab-24-0212-Supplementary-Table-1.pdf]

Supplementary Table 1. The primer information for polymerase chain reactions

**Name**	**Primer sequence**	**Product size (bp)**	**Tm (◦C)**
**VRTN**	F: AAAAGCTCTCCGAAGGAACCC<br>R: GCACCAAGCAGAAGTTATGACC	1351	59
**NR6A1**	F: GAAGTCCCTCCCATTTCCTGTG<br>R: AGCAAGGAAGGATTGGAAGC	223	56
**SYNDIG1L**	F: TCTCCCAGTGACCAGCAAGG R: GCCACCACCACGGCTACAT	133	60
