# Supplementary material for: Understanding molecular mechanisms of vertebral number of variations on Mongolian sheep using candidate genes analysis
Source: Anim Biosci. 2024 Aug 26;38(2):247–54. doi: 10.5713/ab.24.0212 (PMC11725747; doi:10.5713/ab.24.0212)
Supplement: Supplementary file 4 [file ab-24-0212-Supplementary-Table-2.pdf]

Supplementary Table 2. Polymerase chain reactions' conditions for amplification of different genes.

Gene	Initial denaturation	Denaturation	Annealing	Extension	Final extension
VRTN	94 °C for 5 min	94 °C for 45 sec	59°C for 45 sec	72 °C for 90 sec	72°C for 5 min
NR6A1	95 °C for 5 min	95 °C for 10 sec	56°C for 30 sec	72 °C for 40 sec	72°C for 5 min
SYNDIG1L	95 °C for 5 min	94 °C for 10 sec	60°C for 30 sec	72 °C for 45 sec	72°C for 5 min
