# Supplementary material for: Understanding molecular mechanisms of vertebral number of variations on Mongolian sheep using candidate genes analysis
Source: Anim Biosci. 2024 Aug 26;38(2):247–54. doi: 10.5713/ab.24.0212 (PMC11725747; doi:10.5713/ab.24.0212)
Supplement: Supplementary file 5 [file ab-24-0212-Supplementary-Table-3.pdf]

Supplementary Table 3. Descriptive statistics analyses on the body size/body weight in sheep with multiple vertebrae and sheep with normal vertebrae.

**Populations**	**Traits**					
**Normal vertebrae (T13L6)**	BH (cm)		48-85	68.64±0.63	9.93	70
	BL (cm)		53-96	74.99±0.68	9.75	76
	CC (cm)	116	62-110	90.53±0.78	9.27	92
	SW (cm)		5-8	6.47±0.08	12.82	6.5
	BW (kg)		33-84	59.11±0.95	17.32	61
**Multiple vertebrae (T14L7, T14L6, T13L7)**	BH (cm)		53-90	69.59±0.55	8.07	70
	BL (cm)		64-98	79.87±0.74	9.45	80
	CC (cm)	104	78-120	94.81±0.60	6.51	95
	SW (cm)		5-9	6.79±0.07	11.08	7
	BW (kg)		32-101	65.76±0.96	14.83	65

BH, height at withers; BL, body length; CC, chest circumference; SW, shin width; BW, body weight; T13L6, 13 thoracic vertebrae and 6 lumbar vertebrae; T14L7, T14L6, T13L7, 14 thoracic vertebrae and 7 lumbar vertebrae, 14 thoracic vertebrae and 6 lumbar vertebrae, and 13 thoracic vertebrae and 7 lumbar vertebrae (T13L7); CVs, coefficient of variations; mean ± SE, the means with standard errors for traits.
