# Supplementary material for: Understanding molecular mechanisms of vertebral number of variations on Mongolian sheep using candidate genes analysis
Source: Anim Biosci. 2024 Aug 26;38(2):247–54. doi: 10.5713/ab.24.0212 (PMC11725747; doi:10.5713/ab.24.0212)
Supplement: Supplementary file 6 [file ab-24-0212-Supplementary-Table-4.pdf]

Supplementary Table 4. The list of logistic regression models using binomial generalized linear models ran on phenotypic data to check the association associated with the extra vertebrate characteristics from Bayantsagaan sheep in Mongolia.

**Models**	**Degrees of Freedom (DF)**	**AIC**
Extra vertebrate ~ body length	2	284.55
Extra vertebrate ~ body weight	3	306.13
Extra vertebrate ~ body length + weight	3	278.65
Extra vertebrate ~ body length + weight + hearth girth	4	277.05
