# Supplementary material for: Understanding molecular mechanisms of vertebral number of variations on Mongolian sheep using candidate genes analysis
Source: Anim Biosci. 2024 Aug 26;38(2):247–54. doi: 10.5713/ab.24.0212 (PMC11725747; doi:10.5713/ab.24.0212)
Supplement: Supplementary file 7 [file ab-24-0212-Supplementary-Table-5.pdf]

S Table 5. Genetic parameters of candidate loci in Bayantsagaan Mongolian sheep population.

Gene	SNP location	N	Genotype Frequencies			Allele Frequencies		-	Ho	He	PIC
			**CC**	**CT**		**C**	**T**				
**VRTN**	**1712(158)**	145	0.9724 (141)	0.0276 (4)		0.9862	0.0138		0.972	0.027	0.026
	**1716(162)**	145	0.7724 (112)	0.2276 (33)	-	0.8862	0.1138		0.798	0.201	0.181*
	**2067(513)**	141	0.9858(139)	0.0142(2)		0.9929	0.0071		0.985	0.014	0.014
			**GG**	**G/T**	**A/G**	**G**	**T**	**A**			
	**1917(363)**	143	0.9580(137)	0.0140 (2)	0.0280 (4)	0.9790	0.0069	0.0140	0.041	0.958	0.040
			**AA**	**AC**	**CC**	**A**	**C**	**-**			
**NR6A1**	**1523(173)**	207	0.4783(99)	0.3816(79)	0.1401(29)	0.6691	0.3309		0.557	0.442	0.344*

Ho, homozygosity; He, heterozygosity; PIC, polymorphic information content; *PIC>0.5 – Genetic marker is highly polymorphic, with multiple alleles.
