# Supplementary material for: Understanding molecular mechanisms of vertebral number of variations on Mongolian sheep using candidate genes analysis
Source: Anim Biosci. 2024 Aug 26;38(2):247–54. doi: 10.5713/ab.24.0212 (PMC11725747; doi:10.5713/ab.24.0212)
Supplement: Supplementary file 8 [file ab-24-0212-Supplementary-Table-6.pdf]

Supplementary Table 6. The list of logistic regression models run on VRTN loci and the phenotypic data to check the association associated with the extra vertebrate characteristics from Bayantsagaan sheep in Mongolia.

**Models**	**Degrees of freedom (df)**	**AIC**
Extra vertebrae ~ VRTN1716	2	190.55
Extra vertebrae ~ VRTN 1716 + Body Length	3	181.68
Extra vertebrae ~ VRTN 1716 + Body Length + Body Weight	4	180.43
