# Supplementary material for: Understanding molecular mechanisms of vertebral number of variations on Mongolian sheep using candidate genes analysis
Source: Anim Biosci. 2024 Aug 26;38(2):247–54. doi: 10.5713/ab.24.0212 (PMC11725747; doi:10.5713/ab.24.0212)
Supplement: Supplementary file 9 [file ab-24-0212-Supplementary-Table-7.pdf]

Supplementary Table 7. Summary logistic regression results from the top model (Extra Vertebrae ~ VRTN1716 + Body Length + Body Weight) on the VRTN loci and phenotypic variables as additive variables for the association with the extra vertebrate characteristics from Bayantsagaan sheep in Mongolia. The results are calculated using the R package, "gtsummary" (Sjoberg et al., 2021).

**Characteristic**	**Odds Ratio**	**95% Confidence Interval**	**p-value**
**VRTN1716**			
**CC**	-	-	
**CT**	0.24	0.08 – 0.62	0.005**
**Body Length**	1.06	1.01 – 1.12	0.029*
**Body Weight**	1.03	1.00 – 1.07	0.075

* Indicates the statistical significance of p<0.05

** Indicates the statistical significance of p<0.01

Reference for supplementary materials

Sjoberg, D. D., Whiting, K., Curry, M., Lavery, J. A., & Larmarange, J. Reproducible summary tables with the gtsummary package. The R journal, 2021;13(1):570-580. ISSN 2073-4859
